# Ischemic Preconditioning in Atrial Fibrillation

**DOI:** 10.15190/d.2025.12

**Published:** 2025-09-30

**Authors:** Mohamed Ahmed Mohamed, Sankirtana Gorva, Ahmad Hammoud, Shiona Maria Benedict Fernandes, Brandon Sánchez, Pamela González, Srinjoy Dora, Minyoung Kwon, Calista Fernandes, Naga Nandini Mette

**Affiliations:** ^1^David Tvildiani Medical University, Tbilisi, Georgia; ^2^Belagavi institute of medical sciences, Belgaum, Karnataka, India; ^3^Ilia State University, Tbilisi, Georgia.; ^4^Ivane Javaskhilli Tbilisi State University, Tbilisi, Georgia; ^5^Pontificia Universidad Católica Madre y Maestra, Santiago Province, Dominican Republic; ^6^Tbilisi State Medical University, Tbilisi, Georgia; ^7^University of Pennsylvania School of Dental Medicine, Philadelphia, USA; ^8^Georgian National University SEU, Tbilisi, Georgia; ^9^Siddhartha Medical College, Vijayawada, India

**Keywords:** Atrial fibrillation, ischemic preconditioning, myocardial ischemia, ablation.

## Abstract

Atrial fibrillation (AF) is the most common  arrhythmia worldwide with treatments such as  anticoagulants, rate control, and catheter ablation  only aiming to reduce complications. Ischemic  preconditioning (IPC), defined as brief cycles of  ischemia followed by reperfusion, has emerged as a  promising cardioprotective strategy. This review  aims to provide a comprehensive review on and  discuss recent trials surrounding the use of ischemic  preconditioning as a promising therapeutic alternative  in the treatment of atrial fibrillation. Clinical trials in  both surgical and interventional settings have shown  encouraging evidence that these protective  mechanisms can lead to meaningful reductions in  atrial arrhythmia. Despite these encouraging findings,  heterogeneity persists likely due to differences in  patient selection, anesthetic regimens, and the timing  or protocols of preconditioning. The collective  evidence highlights the therapeutic potential  surrounding the use of ischemic preconditioning in atrial fibrillation. With recent trials on the rise, IPC’s  potential impact on rhythm control, pharmacologic  responsiveness, cardioversion outcomes, and  complication rates is highly promising for the  treatment of atrial fibrillation. Further research is  required to translate these findings into routine  clinical practice, particularly in high-risk populations  such as those with concurrent heart failure or  structural heart disease.

## SUMMARY

### 1. Introduction

### 2. Overview of the mechanisms of Ischemic preconditioning

### 3. Overview of atrial fibrillation

### 4. Mechanisms of atrial fibrillation

### 5. Mechanism and the effect of IPC in atrial fibrillation

### 6. Conclusion

## 1. Introduction

Atrial fibrillation (AF) is the most prevalent arrhythmia globally, affecting at least 3 to 6 million people in the United States alone. It arises from abnormal electrical activity in the atria, causing turbulent blood flow that can promote thrombus formation and increase the risk of stroke^1^. AF has various risk factors, with advanced age being the strongest predictor of its development^2^. AF often presents with symptoms such as palpitations and chest pain; however, it may also be asymptomatic in some individuals^2^. Current treatments such as anticoagulants, rate and rhythm control medications and other interventional cardiology procedures substantially help in managing symptoms^1,2^. Despite advances in treatment, sustained rapid electrical impulses may cause structural changes in the atria, thereby increasing the likelihood of recurrence^1^.

Ischemic preconditioning (IPC) is a phenomenon in which tissues undergo a brief period of ischemia followed by reperfusion. Originally discovered by Murry et al. in 1986 in the setting of myocardial ischemia, IPC has since been explored in multiple organ systems^3,4^. IPC has also been extensively investigated in both experimental and clinical settings, consistently demonstrating cardioprotective effects^5,6^.

Given that ischemic, oxidative and inflammatory stress play a significant role in the pathophysiology of AF, IPC may offer therapeutic potential by modulating these processes. Preliminary evidence from human perioperative studies shows that IPC has been associated with antiarrhythmic benefits, including in patients undergoing cardiac surgery^5^. Investigations have also shown a reduction in myocardial injury and potential benefits in patients undergoing catheter ablation for AF^6^.

This review aims to provide a comprehensive evaluation of the available research on the role of ischemic preconditioning in the treatment and management of atrial fibrillation.

## 2. Overview of the mechanisms of Ischemic preconditioning

**Figure 1:**
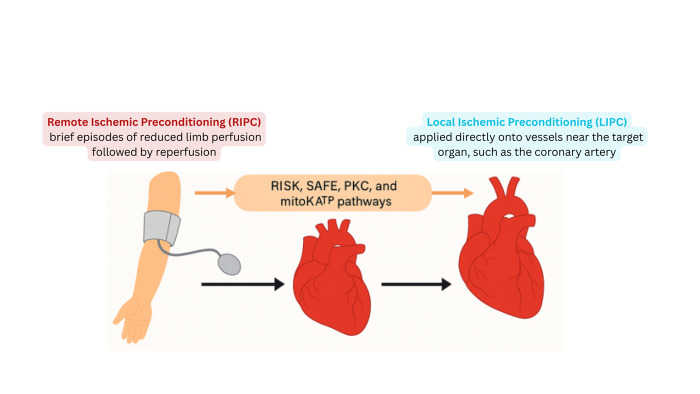
Illustration of remote and local ischemic preconditioning and the mediators involved in both types of ischemic preconditioning.

Ischemic preconditioning is an important mechanism that helps in altering cardiac vulnerability to ischemic injury influencing its response both in physiological and pathological states. It involves inducing brief periods of non-lethal ischemia on cardiomyocytes, activating complex signaling pathways to promote protective effects in the event of subsequent, prolonged ischemia^7^. Ischemic preconditioning can be categorized into two primary types: local ischemic preconditioning (LIPC), which occurs near the heart, and remote ischemic preconditioning (RIPC) (Figure 1).

**Figure 2:**
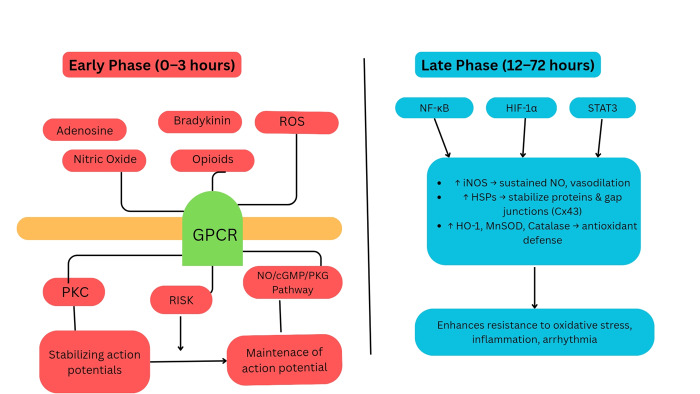
Signaling pathways involved in ischemic preconditioning can be divided into those taking place within the early phase (first few minutes to 3 hours) and late phase (12-72 hours)

RIPC provides a non-invasive, systemic strategy for cardioprotection, whereas LIPC is more intraoperative and site-specific focused^8^. While both activate similar cardioprotective pathways including Protein Kinase C (PKC) activation for tolerance to ischemia, reperfusion injury salvage kinase (RISK) activation, and opening of the mitochondrial ATP sensitive K+ (mitoK-ATP) channel, their mechanisms of initiation differ and different chemical mediators are involved in both the early phase (0-3 hours) and late phase (12-72 hours) after IPC Figure 2^9-11^.

In contrast, remote ischemic preconditioning involves the application of transient, non-lethal ischemia to a distant organ, most frequently a limb, utilizing readily available tools such as a blood pressure cuff. This innovative technique has received the substantial attention for its non-invasive and systemic properties, demonstrating the potential to safeguard vital organs which includes the heart, brain, and kidneys against ischemia-reperfusion injury^12^. From a mechanistic point of view, RIPC starts a cascade of humoral factors, including adenosine, interleukin-10, and stromal-derived factor-1α. Further, it engages extracellular vesicles and neural reflex pathways through the vagus nerve and spinal afferents, eventually converging on key signaling hubs within cardiomyocytes^13^. This comprehensive approach highlights the promising role of RIPC in organ protection and its potential applications in clinical settings.

In healthy physiological states, IPC functions as an adaptive protective mechanism that can be triggered naturally or applied therapeutically. For instance, studies have shown that pre-infarction angina stimulates intrinsic IPC pathways, which leads to reduced infarct size and better clinical outcomes in patients who are experiencing myocardial infarctions^14^. Likewise, regular aerobic exercise may induce a preconditioning-like effect by improving mitochondrial efficiency, nitric oxide bioavailability, and antioxidant capacity. These adaptations help to make the myocardium more resilient to ischemic insults^15^.

The protective effects of ischemic preconditioning are primarily facilitated through early-phase mechanisms involving adenosine, bradykinin, and endogenous opioids as well as late-phase mechanisms mediated by subsequent genomic response that improves the expression of protective proteins, such as antioxidant enzymes, anti-apoptotic proteins, and stress proteins like heat shock protein 70 (HSP70) and heme oxygenase-1 (HO-1)^9-12^. Studies suggest that athletes and individuals who are exposed to intermittent hypoxia, particularly at high altitudes, have evidence of upregulated reperfusion injury salvage kinase (RISK) and survival activating factor enhancement (SAFE) signaling pathways; This supports the findings that exposure may condition the heart for increased resilience against more pronounced ischemic events^8,16^.

The effectiveness of ischemic preconditioning can be significantly compromised in many pathological conditions such as diabetes mellitus, where hyperglycemia alters the activation of the PI3K/Akt signaling pathway which contributes to increased oxidative stress therefore, hindering the mitochondrial K-ATP channel opening which is a vital step in cardioprotection effects of IPC^17^. It’s interesting to note that sulfonylureas like glibenclamide have been shown to inhibit mitoK ATP channels and effectively block IPC’s protective effects^18^.

Moreover, conditions like hypertension, hyperlipidemia, obesity and chronic kidney disease can contribute to chronic inflammation and endothelial dysfunction, which negatively impacts receptor-mediated signaling and affects kinase stream which is critical for IPC^19,20^. Advanced age (which is a significant risk factor for AF) is another factor that can impact IPC response largely due to reduced mitochondrial function, increased basal oxidative stress, and impaired NO signaling^21^.

Several clinical trials, such as ERICCA and RIPHeart have demonstrated varying outcomes that highlight the importance of further classification based on their phenotypic characteristics and the time period between interventions^12,22^.

## 3. Overview of atrial fibrillation

Atrial fibrillation is the most common cardiac arrhythmia worldwide, causing significant morbidity and mortality. The risk for AF increases with age, the lifetime risk of AF is approximately one in 3-5 people. Global prevalence has increased from 33.5 million to 59 million between 2010 and 2019^23^. The 2017 Circulation Research review by Staerk et al. comprehensively analyses population-based cohorts (e.g., Framingham Heart Study) and genetic studies, identifying age as the primary AF risk factor, followed by male sex, obesity, and heart failure, driving atrial remodeling and increasing stroke, thromboembolism, and mortality risks^24^.

Atrial fibrillation is classified by the American Heart Association and American college of Cardiology based on duration and persistence into four categories. Paroxysmal AF is characterized by intermittent episodes that either terminate spontaneously or within 7 days of intervention. Persistent AF, involving episodes sustained beyond 7 days, long standing persistent AF is defined as continuous AF lasting over 12 months and permanent AF, where sustained AF persists and rhythm control strategies are no longer pursued^25^.

The annual progression rate from paroxysmal AF to persistent/afib is 4-5%, modulated by comorbidities such as hypertension, diabetes and heart failure^26^.

10-40% of individuals with AF may be asymptomatic, some manifestations of symptomatic AF may include palpitations, dyspnea, chest discomfort, presyncope, dizziness, reduced exercise tolerance, and fatigue. However, 10% to 40% of individuals with AF may be asymptomatic^27^. AF, particularly in elderly, markedly heightens the risk of thrombus formation, primarily in the left atrial appendage, due to a combination of blood stasis, endothelial dysfunction, and a hyper coagulable state. These clots can embolize and obstruct blood vessels, leading to ischemic events such as strokes, transient ischemic attacks or myocardial infarctions^27,28^. This risk is appreciable even in patients without coronary heart disease, in addition, AF increases the risks of all-cause mortality and heart failure, regardless of history of cardiovascular disease^29^. Patients with AF at moderate to high stroke risk undergoing anticoagulation exhibit an elevated risk of thromboembolic events and poorer survival outcomes when presenting with persistent AF compared to paroxysmal AF^30^. A meta- analysis of 30 cohort studies involving more than 4.3 million individuals to examine the impact of AF on cardiovascular outcomes. The study showed that AF is associated with a significantly higher relative risk of stroke, cardiovascular death, and heart failure in women compared to men^31^.

ECGs have traditionally been the gold standard for diagnosing AF, characterized by an irregularly irregular rhythm with absent P waves and variable PR interval. In paroxysmal AF Hxfolter monitors, which provide 24 - 72 hours ECG recording or extended up to 14 days via patch-based systems, significantly improve AF detection by capturing paroxysmal episodes that may be missed on standard 12- lead ECG^32^. The Fitbit heart study involving 419,000 participants, used smartwatch photoplethysmography (PPG) to detect irregular pulses, with AF confirmed in 34% of notified individuals, demonstrating high positive predictive value for AF detection^33^. Similarly , in an inpatient trial with 102 patients monitored simultaneously with a Holter ECG and an Everion PPG wearable device, AF detection showed high sensitivity (95.2%) and specificity (92.5%)^34^.

Together, these findings highlight that wearable technologies, including smartwatch PPG and wrist worn PPG devices, offer reliable and accurate means for early AF detection^33,34^.

Management of AF involves stroke prevention, rate control and rhythm control. Oral anticoagulation therapy (DOACs) is first line of treatment for stroke prevention based on CHA2DS2-VASc^25^. Left atrial appendage closure (LAAC) is a minimally invasive procedure designed to seal the left atrial appendage, significantly reducing stroke risk in patients with AF, particularly those with contraindications to oral anticoagulation^35^. It serves as an effective alternative to long term anticoagulant therapy. In managing AF, initial treatment often focuses on rate control, using beta blockers, calcium channel blockers, digoxin, or amiodarone to maintain a resting heart rate (<110 bpm). Alternatively, rhythm control aims to restore and maintain normal sinus rhythm through cardioversion or antiarrhythmic drugs such as flecainide, amiodarone. For patients with symptomatic paroxysmal atrial fibrillation (PAF), catheter ablation is often preferred as it reduces progression of the disease and improves quality of life^36^. Cardiovascular risk factors, comorbidities, and unhealthy lifestyle are strongly associated with development of AF. Proactive management of these factors is essential for effective AF treatment.

## 4. Mechanisms of Atrial Fibrillation

It remains unclear exactly what causes and sustains the abnormal fibrillatory activity in atrial fibrillation (AF). However, several mechanisms have been proposed to contribute to the development of AF, which is likely caused by multiple factors^24^. The main mechanisms behind AF involve atrial ectopic electrical activity and reentry circuits, both of which are linked to electrophysiological and structural changes in the atria^37^. In 1998, Haïssaguerre et al. were the first to discover ectopic firing activity coming from myocyte sleeves in the pulmonary vein in individuals with paroxysmal AF. After these ectopic foci were ablated, the AF burden was reduced, demonstrating their role in the pathogenesis of AF^38^. It is now known that pulmonary veins have unique properties that promote reentry and ectopic activity. This abnormal activity is triggered by two factors: the activation of the autonomic nervous system, which makes these cells more excitable, and the abnormal Ca2+ management, generating extra impulses^39^.

Research indicates that there are two types of afterdepolarizations responsible for these spontaneous discharges: early afterdepolarization, which occurs when the cell starts to depolarize before fully recovering from the previous impulse, often caused by a prolonged action potential that enables reactivation of calcium channels and increases inward calcium or sodium currents; and delayed afterdepolarization, which happens when the cell, expected to be at rest, becomes active again, usually resulting from abnormal intracellular calcium overload and spontaneous calcium release from the sarcoplasmic reticulum, activating depolarizing currents that lead to triggered activity^40^.

Another contributing mechanism is substrate heterogeneity, which involves disruption of tissue uniformity. Tissue uniformity is necessary for the synchronized propagation of a normal atrial excitation wave. When a heterogeneous point disrupts this electrical substrate, the atrial excitation wave breaks apart and occasionally rotates^37^. When multiple wave breaks happen sequentially, the electrical wave spreads in a chaotic and disorganized way. Substrate heterogeneity may be related to structural remodeling of the atrial tissue, in which fibrosis plays a significant role. Fibrosis interrupts the continuity of myocardial bundles, interfering with gap junctions between cardiomyocytes. Atrial remodeling and fibrosis are initiated by various pathological stimuli, such as cardiac inflammation, pressure overload, atrial dilation due to hypertension or valvular disease, and myocardial injury^41^. These conditions activate fibroblasts, leading to excessive extracellular matrix deposition and collagen-based scar formation within atrial tissue. This fibrotic remodeling disrupts myocardial fiber continuity and electrical coupling, resulting in slowed conduction and unidirectional blocks that create the substrate for re-entry circuits and chaotic wave propagation characteristic of AF^2^.

Structural remodeling occurs within months when AF persists. Blood stasis in the atria leads to endothelial dysfunction and small injuries to the atrial endocardial lining^42^. These lesions allow innate immune cells to migrate to the endocardium and secrete cytokines such as TNF-α, IL-6, and IL-1β, which damage cardiac cells^42,43^. Within cardiomyocytes, the inflammasome is a complex that detects cellular stress and triggers local inflammation. Activation of the inflammasome promotes caspase-1, leading to the production of pro-inflammatory cytokines like IL-1β, IL-18, and TNF-β^42^. Meanwhile, cardiac fibroblasts, which make up most heart cells, are responsible for maintaining the extracellular matrix and participate in the heart's structural remodeling. Triggered by stress and oxidative damage, fibroblasts activate and transform into myofibroblasts, continuously synthesizing extracellular matrix and depositing type I and type III collagen. This creates a vicious cycle resulting in progressive fibrosis^39^. Fibrosis disrupts normal electrical conduction in the atria by creating scar tissue that slows or blocks impulses, promoting abnormal circuits and ectopic activity^44^. This structural remodeling facilitates the initiation and maintenance of atrial fibrillation. Additionally, AF itself can further stimulate inflammatory responses, forming a self-perpetuating “AF promoting AF” cycle^45^.

Atrial fibrillation reduces ejection fraction and contributes to both the onset and progression of heart failure (HF) through various mechanisms, such as cardiomyopathy caused by sustained tachycardia, an irregular ventricular rate, the absence of effective atrial contraction, functional mitral regurgitation, and activation of the renin–angiotensin–aldosterone system (RAAS)^46^. Both diseases tend to coexist since either can lead to the other. An increased heart rate in AF decreases diastolic filling time and raises left ventricular pressure, which lowers cardiac output. Additionally, irregular heartbeats further decrease cardiac output. The resulting reduction in cardiac output triggers RAAS activation, promoting atrial remodeling and fibrosis development, ultimately leading to heart failure^47^. In heart failure, increased filling pressures dilate the left atrium, causing scarring and eventually fibrosis, which disrupts myocardial bundles. This makes it possible for the reentrant circuits and unidirectional blocks that define AF to form^48^. Therefore, AF and HF have a bidirectional pathophysiological relationship.

## 5. Mechanism and the effect of IPC in atrial fibrillation

Ischemic preconditioning has been shown to reduce the incidence and severity of ischemia-induced ventricular arrhythmias such as ventricular tachycardia (VT) and ventricular fibrillation (VF)^49,50^. For instance, Wu et al. conducted a randomized trial involving 86 CABG patients in which ischemic preconditioning reduced postoperative VF, shortened VF duration, and significantly lowered VT episodes during reperfusion and within 24 hours after surgery^50^. These findings have encouraged investigation into IPC’s role in atrial arrhythmias, particularly atrial fibrillation, where its protective mechanisms may provide clinical benefit. IPC is especially lucrative in the treatment or AF where it’s thought to potentially reverse atrial tissue injury, remodeling, and reduce the thrombotic risk which contributes to the development of a pro-arrhythmic substrate^6,51^. By preserving mitochondrial function, stabilizing calcium homeostasis, modulating ion channel activity, and activating protective signaling pathways, IPC can help reduce atrial stress responses that support the initiation and maintenance of arrhythmia^52^.

One key mechanism here is the preservation of mitochondrial function^53^. For example, RIPC has been shown to maintain mitochondrial respiratory capacity in the right atrium during coronary artery bypass graft (CABG) surgery, preventing disruptions in electrolyte and energy balance that increase arrhythmia risk^53^. Supporting this, Candilio et al. conducted a double-blinded randomized controlled trial in 180 patients undergoing elective on-pump CABG and/or valve surgery and showed that RIPC led to a 54% reduction in new-onset postoperative AF within the first 72 hours after surgery, along with a significant 26% decrease in perioperative myocardial injury^54^. In addition, preclinical work indicates that RIPC prevents the upregulation of microRNA-1 (miR-1), a molecule associated with increased myocardial injury and arrhythmogenesis^53^. Together, these mitochondrial and genetic protective effects provide a reasonable mechanistic explanation for the clinical benefits observed with RIPC.

Another important aspect of IPC’s effect on atrial myocytes is its capacity to reduce myocardial injury and inflammation^6^. In a randomized controlled trial by Han et al., involving 72 patients undergoing radiofrequency catheter ablation (RFCA) for paroxysmal AF, the RIPC group showed notably smaller increases in cTnI, hs-CRP, and IL-6 at 24 and 72 hours compared with the control group^6^. These reductions were accompanied by lower levels of atrial remodeling markers, such as matrix metalloproteinase-9 (MMP-9), and indicators of endothelial damage like von Willebrand factor (vWF), both of which are typically elevated after RFCA^6,51^. Collectively, these results suggest that IPC can effectively modulate atrial stress responses and limit the injury-repair cycles that play a role in AF pathogenesis^6^. In addition to preserving atrial myocyte structure and function, IPC also plays a crucial role in maintaining intracellular ionic balance, particularly calcium homeostasis, which is an essential factor in arrhythmogenesis during ischemia-reperfusion injury^22^. When mitochondrial function is disrupted, electrolyte balance can be disturbed, increasing the risk of cardiac arrhythmias^7,22,53^. Certain microRNAs, such as miR-1 and miR-133, involved in cardiac conduction, affect the ryanodine-receptor-2 (RyR2), potentially leading to RyR2 hyperphosphorylation, which in turn increases spontaneous Ca2+ spark frequency and arrhythmia risk^49^. IPC helps counteract this destabilization of calcium signaling by preserving mitochondrial integrity during ischemia-reperfusion stress, thereby preventing intracellular calcium overload in cardiomyocytes^7,22^. Furthermore, IPC downregulates miR-1 and miR-133, whose upregulation is linked to RyR2 hyperphosphorylation and increased calcium leakage^49,53^. Through these combined effects, IPC stabilizes calcium cycling and reduces one of the fundamental contributors of arrhythmogenesis in the ischemia-reperfusion setting. IPC also modulates ion channels and atrial conduction pathways, further reinforcing its protective effects on AF and perhaps counteracting atrial remodeling in long-standing AF^22,52,55^. A central element of this process involves ATP-sensitive potassium (KATP) channels, where activation of mitochondrial KATP channels helps in cardioprotection via mitochondrial preservation during ischemia-reperfusion stress^22^. Additionally, IPC influences sarcolemmal KATP (sarcKATP) channels, whose dysfunction during ischemia prolongs the effective refractory period in ischemic zones and sensitizes the myocardium to arrhythmias^49^. These changes contribute to conduction abnormalities such as dispersion of atrial refractoriness and conduction delays, which are electrophysiological substrates known to promote AF^52,55^. IPC helps stabilize ion channel function and improves atrial conduction velocity and refractory period uniformity, thereby reducing AF susceptibility^52,55^. These effects were validated clinically in the RIPPAF randomized trial by Kosiuk et al., where RIPC significantly reduced AF inducibility and sustainability in patients with paroxysmal AF undergoing catheter ablation^56^. Electrophysiologically, RIPC reduced atrial effective refractory period dispersion and atrial conduction delays at both proximal and distal coronary sinus, but did not alter PA intervals or overall conduction velocities^56^. These findings support the notion that RIPC can improve atrial electrical properties by reducing conduction delay and refractory period variability, providing a mechanistic basis for its antiarrhythmic effects in AF.

Furthermore, IPC may also enhance the success rate of cardioversion. One of the major limitations of electrical cardioversion is early recurrence of AFib, which is often due to a persistently vulnerable atrial substrate. IPC, by stabilizing myocardial membranes and reducing heterogeneity in atrial refractoriness, may increase the likelihood of successful cardioversion and reduce the rate of relapse into AFib^46^. This concept is supported by the PRECON AF trial (NCT05342220), a prospective randomized study where remote IPC was applied prior to electrical cardioversion in patients with persistent AFib. The trial demonstrated that IPC significantly improved cardioversion success rates and reduced early AF recurrence within 30 days by stabilizing atrial electrophysiological properties^55^.

Clinical studies investigating the role of ischemic preconditioning in atrial and ventricular arrhythmias have revealed encouraging protective effects, though several limitations mitigate the interpretation of these findings. Wu et al. provided early evidence that short cycles of cardioplegic IPC could reduce postoperative VT/VF in CABG patients, demonstrating a clear mechanistic rationale and clinical relevance; however, the relatively small sample size, the high baseline incidence of arrhythmias due to three-vessel disease, and the arbitrarily set time windows for early and delayed IPC phases limit generalizability^50^. Candilio et al. strengthened the evidence with a larger cohort of patients, showing reductions in myocardial injury, postoperative AF, and ICU stay with a multilimb RIPC protocol, yet their findings were limited by challenges like blinding, lack of adjustment for multiple comparisons, and potential bias from concurrent therapies such as intravenous GTN, suggesting the results should be considered ‘hypothesis generating’ ^54^. Han et al. extended the concept by applying RIPC in patients undergoing radiofrequency ablation for paroxysmal AF, demonstrating reductions in inflammatory and myocardial injury markers and a trend toward lower early AF recurrence; nonetheless, the single center design, modest sample size, and limited arrhythmia monitoring raise concerns about selection bias and the possibility of missing asymptomatic AF recurrences ^5,6^. Building on this, Kosiuk et al. provided mechanistic insight into electrophysiological changes, showing that RIPC reduced atrial conduction delay and ERP dispersion, potentially lowering AF inducibility and sustainability, yet the acute study design and focus on short-term preoperative and procedural endpoints restrict conclusions regarding long-term clinical impact^56^. Overall, these studies show the protective ability of IPC/RIPC against arrhythmias, while highlighting the need for larger, multicenter well controlled trials to confirm clinical efficacy, standardize protocols, and clarify mechanistic pathways.

Typical treatment for Afib focuses primarily on rate and rhythm control alongside invasive procedures such as catheter ablation or left atrial appendage closure^57^. Although these strategies aim to reduce thrombotic complications, they don’t treat the underlying atrial injury and remodeling that results in the development of Afib. On the other hand, IPC directly functions to reduce the underlying atrial remodeling and further enhance the effectiveness of antiarrhythmic drugs given in the setting of Afib. As such, while conventional drugs aim to reduce the thromboembolic complications associated with Afib, IPC offers the groundbreaking approach of not only complementing existing therapy but also mitigating the very underlying cause driving the development of Afib^7^.

## 6. Conclusion

Ischemic preconditioning, especially in its remote form, stands out as a promising strategy with antiarrhythmic potential. Through its effects on calcium homeostasis, preserving mitochondrial function, and limiting oxidative stress, IPC offers a new therapeutic approach that reduces the underlying atrial remodeling causing atrial fibrillation. Clinical trials in both surgical and interventional settings have shown encouraging evidence that these protective mechanisms can lead to meaningful reductions in atrial arrhythmia, although results remain variable across studies. This variability highlights the influence of factors such as anesthetic agents, patient heterogeneity, and procedural differences.

Despite the promising physiological insights related to IPC and RIPC, the clinical applications of these strategies still face challenges which includes variability in patient responses based on factors like age, comorbidities, medications, and the type of anesthetics used that could affect the efficacy of IPC. Other problems involve the varying protective outcomes noted in clinical trials of cerebral ischemic preconditioning, indicating the need for more studies to define safe and effective protocols. Ischemic preconditioning itself remains a convincing approach to cardioprotection and neuroprotection, with ongoing developments in clinical applications.

Still, the combination of what we know about IPC’s mechanisms and the supporting clinical evidence suggests it could be a useful strategy for preventing atrial fibrillation. While future large-scale, standardized trials are needed to clarify its role in everyday practice, current evidence supports IPC as a biologically solid and clinically meaningful cardioprotective strategy.

## KEY POINTS

• *Ischemic preconditioning, especially in its remote form, stands out as a promising strategy with antiarrhythmic potential *

*• RIPC has been shown to maintain mitochondrial respiratory capacity in the right atrium, preventing disruptions in electrolyte and energy balance that increase arrhythmia risk*
